# Reducing Vulnerability to Climate Change in Sub-Saharan Africa: The Need for Better Evidence

**DOI:** 10.1371/journal.pmed.1001374

**Published:** 2013-01-22

**Authors:** Nothemba Kula, Andy Haines, Robert Fryatt

**Affiliations:** 1 UK Department for International Development (DFID) - Southern Africa, Pretoria, South Africa; 2 Faculty of Epidemiology and Population Health, London School of Hygiene and Tropical Medicine, London, United Kingdom

## Abstract

Robert Fryatt and colleagues argue for more attention to climate change in sub-Saharan Africa, a region that has contributed the least greenhouse gas emissions to the world's total but is more vulnerable to the impacts of climate change than any other.

**Summary Points**

- Sub-Saharan Africa (SSA) has contributed the least of any world region to the global accumulation of greenhouse gas emissions yet will be more vulnerable to the impacts of climate change than any other.
- Despite the growing awareness of Africa's vulnerability to climate change, there is very little empirical evidence on the health effects of climate change in SSA.
- A cross-disciplinary research agenda needs to be developed to enhance understanding of the health effects of climate change in different settings within SSA linking meteorology, climatology, other relevant sectors and health.
- Adaptive capacity needs to be built through health systems strengthening, and developing more resilient health facilities and supply chains.
- The health dividend of many actions to reduce greenhouse gas emissions needs to be better understood and appreciated by the climate change and public health communities.

## Introduction

Sub-Saharan Africa (SSA) has contributed the least of any world region to the global accumulation of greenhouse gas emissions; however, this region will probably be more vulnerable to the impacts of climate change than any other [1]. Less than 7% of the world's total emissions of greenhouse gases emanate from the African continent [2]; thus the continent contributes very little overall to climate change. Yet the highest regional burden of climate change is likely to be borne by SSA, with 34% of the global disability adjusted life years (DALYs) attributable to the effects of climate change in the region [3]. The impacts of climate change are likely to be concentrated in low-income countries where poor populations already have compromised heath prospects, health systems are weak, and where the capacity to adapt and address vulnerabilities is limited. However, to mount a proper response there are some fundamental constraints that need to be addressed, which we further discuss in this Essay.

## The Limited Evidence On Climate Change And Health In Sub-Saharan Africa

Despite the growing awareness of SSA's vulnerability to climate change, there is very little empirical evidence published about the effects of climate change on population health in SSA [4]. Some attention has been given to potential increased incidence of vector-borne diseases such as malaria, particularly in highland areas in East Africa [5],[6]. Although this finding has been contested, increased temperatures together with changes in precipitation may exacerbate malaria epidemics and lead to the spread of malaria beyond its normal transmission zone [7]. Overall, though, any possible effect of climate change on malaria is likely to be two orders of magnitude smaller than those that can be achieved by the effective scale-up of key control measures [8]. It is likely that climate change will alter the ecology and transmission patterns of climate-sensitive, parasitic infections such as schistomiasis, trypanosomiasis, and leishmaniasis, and other diseases such as cholera, diarrhoeal infections, and Rift Valley fever [9]; but the overall impacts on health are unclear.

More significantly, climate change may impact seriously on environmentally sensitive sectors such as water, agriculture, and food production and adversely affect human health and vulnerability to disease as a result [10]. Changing temperatures, humidity, and precipitation are expected to disrupt agricultural production systems, reducing food production and leading to higher levels of malnutrition, which in turn can increase vulnerability to disease. Significant rainfall reductions observed in critical crop-growing areas in East Africa have caused severe drought, resulting in loss of agricultural productivity and famine [11]. Countries like Botswana that have already experienced frequent droughts have seen a large number of people abandoning crop production and moving either to cities or informal settlements [7],[11]. These changes now need to be better understood, with more rigorous assessments and building of evidence.

## Countries In The Region Are Ill-Prepared To Cope With Climate Change

Although African governments have made firm commitments at various forums to adapt to climate change, many are still considered ill-prepared to cope with the negative consequences of climate variability and change [12]. An Action Plan has been prepared [13] covering the following elements: baseline risk and capacity assessments; capacity building; integrated environment and health surveillance; awareness raising and social mobilization; public health oriented environmental management; scaling up of existing public health interventions; strengthening of partnerships; and promotion of research. However, more investment is required if these proposals are to become reality across all countries in SSA.

The United Nations Framework Convention on Climate Change (UNFCCC) initiated National Adaptation Programs of Action (NAPAs) in 2002 in least developed countries to help prioritise adaptation activities. Of the 47 NAPAS submitted to UNFCC as of January 2012, 33 (70%) were from SSA countries and 15 (45%) of these list health as a priority. Table 1 groups health sector adaption projects from those SSA countries that listed health as a priority; this grouping shows that the most frequently listed projects are malaria control, waterborne disease control, health systems strengthening, improved access to safe drinking water and sanitation, as well as establishment of health surveillance and early warning systems for disaster preparedness and response. However, a World Health Organization (WHO) assessment of these interventions concluded that only a few of them were likely to be effective [14].

**Table 1:** Health adaptation strategies proposed by sub-Saharan African countries in their national adaptation programs of action (January 2012).

Adaptation Strategy	Country														
	Benin	Comoros	Central African Republic	Ethiopia	Gambia	Madagascar	Mali	Niger	Sao Tome & Principe	Sierra Leone	Tanzania	Togo	Tuvalu	Uganda	Zambia
1. Protection against malaria	X	X	—	X	—	—	—	—	—	X	X	—	—	—	—
2. Prevention against water-borne diseases	—	—	X	—	—	—	—	—	—	—	—	—	—	—	—
3. Support for eye medical and surgical care	—	X	—	—	—	—	—	—	—	—	—	—	—	—	—
4. Reduction of climate change related diseases	—	—	—	—	X	—	—	X	—	—	—	—	—	—	—
5. Education, awareness raising, and community mobilization	—	—	—	—	—	X	—	—	—	—	—	—	—	—	—
6. Capacity building for the health system	—	—	—	—	—	X	—	—	X	—	—	—	—	—	—
7. Implementation of information systems/databases on climate change risk-related diseases	—	—	—	—	—	—	X	—	X	—	—	—	—	—	—
8. Monitoring and control of water and Sanitation services	—	—	—	—	—	—	—	—	—	X	—	—	—	—	X
9. Monitoring and control of HIV/AIDS prevention activities	—	—	—	—	—	—	—	—	—	X	—	—	—	—	—
10. Prevent and fight vector-borne disease/pests	—	—	—	—	—	—	—	—	—	—	—	X	X	X	—

There are also several multi-country projects that specifically aim to support adaptation within the region; however, a recent review of current and planned adaptation actions [15] concluded that “None of the regional projects identified has as strong focus on freshwater resources, forestry and human health.”

When funds are mobilised across all sectors (summarised in Table 2]), the gap between funding approved and funding disbursed suggests bottlenecks in program implementation [16]. Although the Least Developed Countries Fund (LDCF) has approved the largest volume of adaptation finance for SSA to date, the funding is unevenly distributed across sectors. Table 3 shows that only 4% of funds are allocated to the health sector [17].

**Table 2:** Contributions of dedicated climate funds monitored by CFU to adaptation in sub-Saharan Africa [16].

Climate Funds	Amount Approved (US$ million)	Amount Disbursed (US$ million)	Projects Disbursed
Global Climate Change Alliance	52	22	5
International Climate initiative	12	12	5
Adaptation fund	15	4	2
Least developed countries fund	90	60	49
Special climate change fund	20	12	6
MDG Fund	16	13	3
Pilot program for climate resilience	114	0	0
Total of SSA	**319**	**123**	**70**

**Table 3:** Prioritization of funding in least developed countries [23].

Development Sectors Prioritized in NAPAs	Percent
Food security and agriculture	28
Water resources	14
Early warning and disaster risks	16
Coastal management	15
Ecosystems	15
Capacity building	6
Health	4
Energy	2

## More Urgent Action Is Required To Build Capacity To Respond To Climate Change And Variability

### Building An Evidence Base

A review of health sector policy options for health adaptation [18] lists a range of specific actions that the health sector should undertake to reduce cases of disease and deaths attributable to climate change (Box 1). For example, SSA countries need to establish the vulnerability of existing populations to climate-sensitive health risks [12] and plan adaptation on the basis of detailed assessments of national vulnerabilities to specific health risks [19]. Research infrastructure in many African settings needs to be improved and investments need to be made to strengthen networks of institutions to conduct cross-disciplinary work between meteorology/climatology, other relevant sectors and health, building on what has already started [20]. Research is required to enhance understanding of the health effects of climate change in different settings within SSA and to generate and disseminate knowledge on appropriate local adaptation measures.

**Box 1. Examples Of Health Sector Policy Options For Adaptation To Climate Change [17]**

- Improving, modifying, or expanding health protection systems including surveillance systems for vector- and water-borne diseases and, seasonal forecasting and early warning systems for infectious diseases (e.g., epidemic malaria).
- Developing and implementing health forecasting and early warning systems (including emergency incident response plans) for extreme events such as heat- and flood-health warning measures.
- Maintaining and improving current environmental health regulatory standards (e.g., water and air quality standards).
- Improving or modifying health systems infrastructure by adapting hospitals and clinics to increased frequency of extreme weather events such as heat waves and floods.
- Increasing the capacity of health care services and human resources to cope with additional disease burden associated with extreme weather events.
- Preventing or treating the additional cases of diseases due to failure in adaptation upstream.
- Improving the provision of medication to reduce the impact of potential increases in infectious disease transmission.

### Keeping A Focus On Health And Health Systems

A range of empirical studies demonstrate that advancing broader development goals is a crucial pre-requisite for an effective climate change response [21]. WHO suggests that health-system actions that can also protect populations from the impacts of climate change should encompass public health interventions, such as control of neglected tropical diseases and provision of primary health care, and actions to address the environmental and social determinants of health [19]. There is a strong case for prioritising these factors that determine how well the health system will respond to impacts of climate on health. For example it will be important to consider how health facilities and supply chains may be affected by climate extremes—floods, storms, droughts, and heat waves—and how they can be made more resilient.

### Promoting The Health Benefits Of Low Carbon Strategies

“Health co-benefits” is the term used to describe the ancillary benefits to health as a result of climate change mitigation strategies [22]. There are significant win-win options that can reduce greenhouse gas emissions as well as contribute to better health. Many measures to reduce greenhouse gas emissions in household energy, transport, food, and agriculture and electricity generation have substantial health benefits [23]. To achieve mitigation with a health dividend, policy makers will need to prioritize increased active transport (walking and cycling) public transport systems and reduced private-car use in urban settings, increased uptake of improved cooking stoves in low-income countries, reduced consumption of animal products in high-consumption settings, and generation of electricity from affordable, clean, “low-carbon” sources, as well as avoidance of highly polluting biomass [24]. These strategies can address major health problems such as child mortality from acute respiratory infections, ischaemic heart disease in adults, and other non-communicable diseases such as obesity, diabetes, and depression.

### Increase Funding For Work On Climate Change And Health

With less than 4% of funds currently allocated to health protection, and given the region's vulnerability, there is a compelling case for SSA to receive significant funding for additional health adaptation. This should be targeted at building a stronger evidence base for future policy makers and documenting specific actions that will enhance adaptation to climate change together with low carbon strategies that will also benefit health.

## Conclusions

There is a need to rapidly build the adaptive capacity in existing health institutions in SSA, and to develop a stronger evidence base for local adaptation strategies in vulnerable sectors such as health. Such action will also assist the region to successfully access additional adaptation funding that may be available. The health sector needs to engage in partnerships with other organizations and sectors to ensure that health concerns are adequately integrated into the work of national climate change adaptation committees, National Adaptation Programmes of Action, and regional and international adaptation and mitigation strategies. This collaboration should include more recognition of the potential ancillary benefits to health from low carbon development.

## Abbreviations

DALY: disability adjusted life year
LDCF: Least Developed Countries Fund
NAPA: National Adaptation Programs of Action
SSA: sub-Saharan Africa
UNFCCC: United Nations Framework Convention on Climate Change
WHO: World Health Organization
